# Research on the health of people who experience detention or incarceration in Canada: a scoping review

**DOI:** 10.1186/s12889-015-1758-6

**Published:** 2015-04-25

**Authors:** Fiona G Kouyoumdjian, Andrée Schuler, Stephen W Hwang, Flora I Matheson

**Affiliations:** Centre for Research on Inner City Health, St. Michael’s Hospital, Toronto, ON Canada; Centre for Research on Inner City Health and Department of Medicine, St. Michael’s Hospital, Toronto, Canada

**Keywords:** Prisoners, Health status, Canada, Review

## Abstract

**Background:**

We conducted a scoping review to define the extent and type of quantitative health status research conducted from 1993 to 2014 with people who have experienced detention or incarceration in correctional facilities in Canada.

**Methods:**

We searched 15 databases, reviewed reference lists and relevant websites, and consulted with key stakeholders to identify eligible studies. We reviewed records for eligibility and extracted relevant data from eligible articles.

**Results:**

We identified 194 studies that were eligible for inclusion. Most studies were conducted with males and with persons in federal facilities, and focused on mental health, substance use, and social determinant of health outcomes.

**Conclusions:**

Health status data are limited for several outcomes, such as chronic disease, injury and sexual and reproductive health, and for persons in provincial facilities and post-release. Efforts should be made to improve data collection and knowledge dissemination, so that relevant data can be used more effectively to improve health and health care in this population.

**Electronic supplementary material:**

The online version of this article (doi:10.1186/s12889-015-1758-6) contains supplementary material, which is available to authorized users.

## Background

Worldwide, more than 11 million people are imprisoned at any given time, and more than 30 million people move through the prison system annually [1,2]. In Canada, there are more than 250,000 adult admissions and 14,000 youth admissions each year to correctional facilities [3,4]. On any given day, there are about 40,000 adults and youths in correctional facilities [5-7].

In Canada, jurisdiction over correctional facilities is shared between the federal, provincial, and territorial governments. Admission to a correctional facility prior to sentencing is called remand, and persons in remand are considered detained rather than incarcerated. Persons in remand and persons who are sentenced to less than 2 years are detained or incarcerated in provincial or territorial facilities, and those who are sentenced to 2 years or longer are incarcerated in federal facilities.

International data suggest that people who experience detention or incarceration have poor health compared with the general population, as indicated by data on the prevalence of mental illness, infectious diseases, chronic diseases, and mortality [8]. Detention and incarceration may serve as a unique opportunity to provide health care, to initiate programs to improve health, and to link persons with appropriate services on release. Such interventions could improve the health of people who experience detention and incarceration, and also decrease health care costs [9], improve health in the general population [9-14], improve public safety [9], and decrease re-incarceration [9,15,16]. Decisions regarding priorities for research, programs and policies should be informed by Canadian data on the burden of disease and interventions in this population [17].

We conducted a scoping review to describe the extent and type of quantitative health status research conducted between 1993 and 2014 on people who have experienced detention or incarceration in correctional facilities in Canada. In summarizing these data, we aimed to identify areas that have been well defined and gaps in evidence that we can use to inform future research.

## Methods

We conducted the scoping review [18,19] according to a protocol that we defined *a priori* (available from authors on request).

### Search strategy

We searched Medline, PsycINFO, Embase, the Cochrane Library, Social Sciences Abstracts, Social Services Abstracts, Sociological Abstracts, CINAHL, Criminal Justice Abstracts, ERIC, Proquest Criminal Justice, Proquest Dissertations and Theses, Proquest Dissertations and Theses: UK and Ireland, Web of Science, and Scopus in April 2014 (see Additional file 1 for search strategy). We reviewed reference lists of included studies and relevant reviews. We did not use any language restrictions, though we used only English language search terms. We searched websites of relevant organizations, specifically the Correctional Service of Canada, Statistics Canada, the Office of the Correctional Investigator, Public Safety Canada, the provincial and territorial Ministries responsible for correctional facilities, PASAN, The John Howard Society of Canada, the Canadian Association of Elizabeth Fry Societies, and the Canadian HIV/AIDS Legal Network. We also consulted with knowledgeable persons in some of these organizations.

### Study selection and data extraction

#### Population

We included studies of adults and adolescents who had been detained or incarcerated in a prison or jail in Canada, whether they were remanded or sentenced, and whether the study focused on the period prior to, during, or subsequent to detention or incarceration. We included studies that included other populations if the studies presented stratified results for persons who met this population criterion. We excluded studies that did not specify that participants had been in detention or incarcerated, *e.g.* studies of “offenders” or “forensic” populations that did not specify a history of detention or incarceration.

#### Study period

Studies were eligible if they reported data on health from 1993 to 2014. We chose 1993 as the start date for our period of eligibility because we wanted to capture recent data and we hypothesized that a reasonable number of studies would have been conducted after this date. In addition, this date follows the enactment of the federal Corrections and Conditional Release Act in 1992 [20], which may have affected the health care services provided in federal correctional facilities. We included studies that did not specify the dates on which they were conducted if they were published in or after 1997, which assumes a maximum four-year lag time from conducting a study to publication.

#### Study types

We included experimental studies (*i.e.* randomized controlled trials, quasi- randomized controlled trials, and non-randomized controlled trials), quasi-experimental studies (*e.g.* controlled before-after studies, interrupted time series studies), and observational studies (*i.e.* cohort studies, case–control studies, cross-sectional studies). We included reports of administrative data as well as studies that collected primary data.

#### Outcomes of interest

We included indicators of health as defined by the Canadian Institutes for Health Information [21], including indicators of health status and health system performance, and social determinants of health as per the Public Health Agency of Canada [22]. Since we were interested in defining health status, we included studies that provided absolute measures of health (*e.g.* percent or number of persons with a certain condition or behaviour) and not studies that specified only relative measures of health (*e.g.* relative risks, odds ratios, *etc*.). For some determinants of health, such as gender, race/ethnicity, employment and education status, we included studies only if they also specified other health status data or if they summarized these data for the whole source population of interest. We included only studies that reported individual-level data (*e.g.* a study that reported only the total proportion of urinalysis tests that were positive in an institution but did not specify the number or percent of persons who had positive tests would be excluded).

#### Review procedures

Two reviewers (FGK and AS) independently reviewed titles and abstracts for eligibility, and any disagreements were resolved by discussion. For full article review, we conducted a pilot to ensure a high level of agreement regarding eligibility. One reviewer (FGK or AS) then reviewed each full article to assess eligibility, with discussions regarding any decisions that were not clear.

#### Data extraction

For eligible articles, one reviewer (FGK or AS) extracted data using a data extraction form that we developed, piloted, and modified. We extracted data on study context, populations included, design, outcomes, and results.

#### Analysis

We planned *a priori* to summarize data based on study design, whether each study was conducted in the federal or provincial/territorial system, with females or males, and with youth or adults, in which provinces or territories the study was conducted, and the types of outcomes included.

We classified observational studies as cross-sectional if the data were collected at a single point in time and no intervention occurred, and longitudinal if the data were collected over a period of time and no intervention occurred. We categorized interventional studies as per the Cochrane Handbook classification (Table 13.2) [23]. We classified a study as involving youth if the record specified that youth or adolescents were involved or that the study was conducted in a facility for youth or adolescents or with persons younger than 20, and as involving adults if the record specified that the study was conducted with adults or in a facility for adults, or if persons included were aged 17 and older. We categorized outcomes and behaviours into one of the following categories: death, chronic disease, communicable disease, mental health excluding substance use outcomes (using DSM-V categories when appropriate [24]), substance use, injury, sexual and reproductive health, health system, and social determinants of health.

To identify data from studies that were reported in multiple publications, we sorted and compared extracted data on the basis of author name, study location, dates, and sample size. For the purposes of summarizing data, we considered data described in multiple publications as a single study if the sample data matched.

## Results

**Figure 1:**
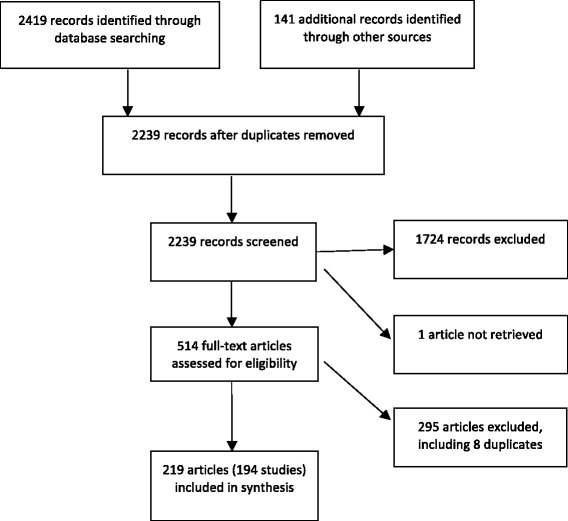
Flow diagram of study selection.

As shown in Figure 1, we identified 2560 records: 2419 through database searches, 34 from reference lists and sources known to the authors, and 107 on websites. After eliminating duplicates, there were 2239 records remaining. Of these, 515 were eligible for full review. We were unable to retrieve 1 article [25]. Of the remaining 514 full articles, 8 were duplicates that had not been identified previously, and 219 articles were eligible for inclusion. These 219 articles represent 194 unique studies (see Additional file 1).

Key characteristics of included studies are summarized in Table 1. Seventy-five studies were conducted in multiple provinces, of which 92.0% (n = 69) were conducted in federal correctional facilities only. One third of studies were conducted in British Columbia, Ontario, or Quebec, and in almost ten percent of studies, the geographical location was not specified. More than 60% of studies were conducted in only federal facilities. Over ninety percent of studies were conducted with persons while detained or incarcerated, and collected data only from the period while they were detained or incarcerated. Eighty-six studies included only men, and in another 35 studies, more than two thirds of participants were men. More than three quarters of studies included only adults. Seventy-one point six percent of studies were cross-sectional, 20.6% were longitudinal, and 7.7% were interventional.

**Table 1:** **Characteristics of studies conducted from 1993 to 2014 on the health status of people who have experienced detention in Canada, N = 194**

**Characteristic**		**Number (%) of studies**
**Geographic location**	Alberta	2 (1.0)
	British Columbia	29 (14.9)
	Manitoba	3 (1.5)
	New Brunswick	1 (0.5)
	Newfoundland and Labrador	1 (0.5)
	Nova Scotia	1 (0.5)
	Ontario	44 (22.7)
	Quebec	17 (8.8)
	Saskatchewan	3 (1.5)
	multiple provinces	75 (38.7)
	not specified	18 (9.3)
**Conducted in federal or provincial facilities**	federal	121 (62.4)
	provincial	48 (24.7)
	both	20 (10.3)
	not specified	5 (2.6)
**Population included**	persons in correctional facilities	183 (94.3)
	persons after release	6 (0.03)
	both	5 (0.03)
**Period of data collection**	while in correctional facilities only	182 (93.8)
	prior to admission to or subsequent to release from correctional facilities	6 (3.1)
	both	6 (3.1)
**Participant gender**	male	86 (44.3)
	female	3 (1.5)
	both	67 (34.5)
	not specified	10 (5.2)
**Participant age group**	adults	162 (83.5)
	youth	24 (12.4)
	both	6 (3.1)
	not specified	2 (1.0)
Study design*	intervention	15 (7.7)
	randomized trial	2 (1.0)
	controlled before-after study	4 (2.1)
	before-after study	7 (3.6)
	cross-sectional	2 (1.0)
	longitudinal	40 (20.6)
	cross-sectional	139 (71.6)

*For included data.

The majority of studies (N = 119) were conducted with the general inmate population, with some exclusions based on concerns about language, literacy, safety, or mental health. Other populations studied include persons who were participating in specific programs in correctional facilities (N = 17), persons convicted of sexual offenses (N = 12), persons who used drugs (N = 5), Aboriginal persons (N = 5), and persons with a history of self-injury (N = 5). One quarter of studies (N = 49) included less than 100 participants, 17 of which were conducted in the general population.

Sixty-four studies used only data from administrative databases or file reviews, *i.e.* the researchers did not collect primary data, most of which (n = 58) were conducted only in federal facilities. Administrative databases used in studies of persons in federal detention include the Offender Management System, Situation Reports, Offender Intake Assessment, the Computerized Mental Health Intake Screening System, Computerized Lifestyle Assessment Instrument, Computerized Assessment of Substance Abuse, Infectious Diseases Surveillance System, Tuberculosis Tracking System, and Canadian Police Information Centre. Administrative databases used in studies of persons in provincial facilities include the British Columbia Cervical Cancer Screening Program and the British Columbia Medical Services Plan.

For 65 studies, the dates when the study was conducted were not clearly specified. Dividing the period under study (1993 to 2014) into two equal intervals, it was possible to ascertain the period when the study was conducted for 154 studies based on the dates of publication as well as the dates when the study was conducted as reported in articles; data were collected before 2004 in 80 studies, between 2004 and 2014 in 51 studies, and spanning both periods for 23 studies.

Table 2 shows the number of studies that reported outcomes in different health status categories. A large number of studies reported mental health (N = 99), substance use (N = 86), and social determinants of health (N = 80) outcomes, while comparatively few studies reported communicable disease (N = 35), injury (N = 30), sexual and reproductive health (N = 29), and in particular chronic disease (N = 6) outcomes. Regarding specific outcomes, a large number of studies reported data on mental disorders (N = 79) such as personality disorders (N = 37), bipolar and related and depressive disorders (N = 31), anxiety and obsessive-compulsive and related disorders (N = 19), and schizophrenia spectrum and other psychotic disorders (N = 16); substance related disorders (N = 48) and substance use (N = 44); race, ethnicity, or Aboriginal status (N = 33); experiences of abuse in childhood and adulthood (N = 29); self-injury (N = 29) including self-harm and suicide attempts; and bloodborne or sexually transmitted infections (N = 28).

**Table 2:** **Outcomes reported in studies conducted from 1993 to 2014 on the health status of people who have experienced detention in Canada, N = 194**

**Outcomes reported by category**	**Number of studies**
**Deaths**	14
Suicide	6
**Chronic disease**	6
Fetal alcohol spectrum disorder	4
Epilepsy	2
Cardiovascular disease	1
Respiratory disease	1
Diabetes	1
Urological disorder	1
**Communicable diseases**	35
Bloodborne or sexually transmitted infections	28
Unsafe injection equipment for drug use, tattooing or piercing	11
Tuberculosis prevalence	2
Immunization status	2
**Mental health**	99
Mental disorders	79
Intellectual disabilities	6
Attention deficit hyperactivity disorder	5
Specific learning disorder	4
Schizophrenia spectrum and other psychotic disorders	16
Bipolar and related and depressive disorders	31
Anxiety and obsessive-compulsive and related disorders	19
Trauma- and stressor-related disorders	10
Dissociative disorders	2
Somatic symptom disorders	3
Disruptive, impulse control and conduct disorders	4
Eating disorders	1
Gambling disorder	3
Personality disorders	37
Paraphilic disorders	3
Suicidality	21
Quality of life	1
Gambling	3
Psychiatric symptoms	26
Alexithymia	2
**Substance use**	86
Substance-related disorders	48
Substance use other than smoking	44
Smoking	7
Injection drug use	22
Overdose	2
Driving after substance use	3
**Injury**	30
Self-injury	29
Suicide attempt	19
Head injury/traumatic brain injury	3
**Sexual and reproductive health**	29
Sexual behaviours	16
Number of partners	5
Same sex partners	6
Condom use	13
Contraceptive use	1
Pregnancy history	5
Parity	7
Sex trade involvement	14
Cervical cancer screening results	3
**Health system**	62
Accessibility	20
Health care utilization	18
Hospitalization	10
Outpatient/clinic services	7
Dentist	2
Appropriateness	32
Screening	18
Mental health	3
Fetal alcohol spectrum disorder	1
Cancer	1
Latent tuberculosis	1
Bloodborne infections: HIV and hepatitis C	11
Treatment	20
HIV treatment	2
Hepatitis C treatment	4
Latent tuberculosis treatment	1
Substance abuse treatment	7
Methadone maintenance treatment	5
Medication prescribed	6
Acceptability	22
Program participation	5
Program/treatment completion	8
Program/treatment satisfaction	5
Medication usage	6
Medication adherence	2
Effectiveness	8
Health needs	8
**Social determinants of health**	80
Race/ethnicity/Aboriginal status	33
Religion	1
Employment	10
Education	7
Income	6
Sexual orientation	4
Language	5
Housing	13
Experienced discrimination	2
Abuse	29
Childhood abuse	25
Other adverse childhood experiences	16
Child welfare involvement	9
Out of home placement	4
Residential school involvement	4
Witnessed domestic violence	9
Family and social connectedness	18
Coping skills	4
Locus of control	3
Self-esteem	6
Intelligence	2
Literacy	1

## Discussion

This review identified 219 publications representing 194 studies that were conducted from 1993 to 2014 with people who experienced detention or incarceration in correctional facilities in Canada. The majority of studies were conducted with persons during detention or incarceration, with persons in federal facilities, and with only men. The greatest number of studies presented mental health, substance use and social determinant of health outcomes, and few studies reported chronic disease, sexual and reproductive health, and injury outcomes.

Notably, the great majority of people who experience detention or incarceration in Canada have short sentences or are in remand and therefore serve their time in provincial or territorial facilities, with less than 5% of admissions to federal facilities [3,4,26]. This review found that most research on health status conducted from 1993 to 2014 has focused on persons in federal facilities. This may be due to longer periods of incarceration in federal facilities that provide more time to assess health and conduct research, a greater focus on rehabilitation (including health) in the federal system, greater accessibility of administrative and health data in the federal system compared to provincial facilities, or the challenges of following persons post-release who are not under community supervision. More clearly defining health and intervening to improve health in persons who experience detention or incarceration in provincial and territorial facilities could have a relatively large health impact on this population, with potential ripple effects on family and community health, public safety, and costs to society of health care and criminal justice system involvement [9].

Considering the large number of people who experience detention and incarceration each year in Canada and the fact that the State has a clear obligation to provide health care during detention and incarceration, the number of studies identified in this review is small. There is a particular lack of data in this population for important outcomes such as chronic diseases, sexual and reproductive health, and injury, some of which may be amenable to primary, secondary, and tertiary prevention interventions [27]. The paucity of evidence identified could reflect a lack of collection of health data (whether routinely or for specific projects), a failure to analyse collected data or to disseminate collected data, or limitations in our search strategy. It may also be, in part, a downstream effect of the lack of dedicated funding in Canada for research focused on prison populations, in contrast with the USA [28].

Population health status data should inform decisions about how to focus limited resources in correctional facilities and after release, and data on the general population of persons who experience detention and incarceration could be used to determine the population burden and to estimate and compare the impact of proposed interventions. Instead of multiple independent small studies of specific outcomes, researchers and health administrators should use strategies that are more efficient and provide a more complete picture. Existing processes at intake or routine evaluation could be optimized by identifying which data should be routinely collected [29], standardizing questions or measurements across jurisdictions including levels of government, and implementing or improving the use of electronic databases [30]. A periodic population health survey could provide a cross-section of health data across persons in detention in Canada, potentially including physical measurements and biological sampling [31]; similar comprehensive health surveys have been conducted across correctional facilities in the USA [32,33] and in federal facilities in Canada [29,34-40]. Further, with appropriate measures in place to ensure informed consent and privacy, data from administrative sources and from periodic surveys could be linked to external administrative data sources to look at health status in the community before and after detention or incarceration, including vital statistics registries, health services utilization data, and social services utilization data [41,42].

There are several potential limitations to this review. We included only studies that reported absolute data on health status, given our interest in understanding the quantitative burden of disease, which means that we have excluded some studies that reported health outcomes, and we did not include qualitative data. While we endeavoured to optimize our search strategy, we may have missed relevant studies, as noted above, especially studies that were not published in the peer-reviewed literature. We have described our search strategy in detail and provided our search terms in an Additional file 1 for transparency and reproducibility. Though we aimed to capture studies conducted from 1993 through 2014, our study would not have captured most studies conducted in the past few years, given the typical lag in time from data collection to study publication. Regarding study procedures, only one author reviewed most full articles and extracted data from eligible articles, which may have led to errors in determination of eligibility or in the data presented. We attempted to minimize errors by discussing and defining in detail the eligibility criteria, piloting our review and data extraction process to achieve a high level of consistency and accuracy, and checking extracted data.

More work is required to improve our knowledge about the health of persons who experience detention and incarceration in Canada and to facilitate the application of health status data. Together with key stakeholders, including persons with a history of detention or incarceration [43], provincial, territorial and federal governments should consider which health status data are required for action to improve health, health care and security, and then ensure that these data needs are reflected in data collection at intake and other routine evaluations, as well as in surveillance programs, surveys, and other research initiatives. Consideration should be given to the analysis and dissemination of collected data to optimize their reach and impact. Persons conducting research and making decisions about health initiatives need to review both published and gray literature to inform their work and to minimize the duplication of research efforts [17]. Finally, as data emerge on various aspects of health in this population and on effective interventions, we should iteratively assess and define research priorities for improving health.

## Conclusions

Health status data are limited for persons who experience detention and incarceration in Canada. Data are lacking on chronic disease, injury and sexual and reproductive health outcomes, and for persons in provincial facilities and after release. Further research should be done to elucidate health status in this population, and research should be streamlined to improve efficiency. Consideration should be given to which data are required for action to improve health and health care in this population, and efforts should be made to ensure that knowledge is disseminated to decision makers and other key stakeholders.

## Additional file

Additional file 1:
**Search strategy and reference list for identified studies.**

## References

[1] Walmsley R. World prison population list, 10th edition. London: King's College London International Centre for Prison Studies, 2013.

[2] Kinner SA, Forsyth S, Williams G (2013). Systematic review of record linkage studies of mortality in ex-prisoners: why (good) methods matter. Addiction.

[3] Perrault S. Admissions to adult correctional services in Canada, 2011/2012. http://www.statcan.gc.ca/pub/85-002-x/2014001/article/11918-eng.htm - a2. 2014. Accessed December 14 2014.

[4] Perrault S. Admissions to youth correctional services in Canada, 2011/2012. http://www.statcan.gc.ca/pub/85-002-x/2014001/article/11917-eng.htm?fpv=2693. 2014. Accessed September 22 2014.

[5] Statistics Canada. CANSIM Table 251–0005: Adult correctional services, average counts of offenders in provincial and territorial programs: Annual. http://www5.statcan.gc.ca/cansim/a05?lang=eng&id=2510005&pattern=2510005&searchTypeByValue=1&p2=35. 2014. Accessed September 22 2014.

[6] Statistics Canada. CANSIM Table 251–0006: adult correctional services, average counts of offenders in federal programs: Annual. http://www5.statcan.gc.ca/cansim/a05?lang=eng&id=2510006&pattern=2510006&searchTypeByValue=1&p2=35. 2014. Accessed September 22 2014.

[7] Statistics Canada. Youth correctional services, average counts of young persons in provincial and territorial correctional services: Annual (persons). http://www5.statcan.gc.ca/cansim/a05?lang=eng&id=2510008&pattern=2510008&searchTypeByValue=1&p2=35. 2014. Accessed September 22 2014

[8] Fazel S, Baillargeon J (2011). The health of prisoners. Lancet.

[9] Kinner S, Wang EA (2014). The case for improving the health of ex-prisoners. Am J Public Health.

[10] Lee H, Wildeman C, Wang EA, Matusko N, Jackson JS (2014). A heavy burden: the cardiovascular health consequences of having a family member incarcerated. Am J Public Health.

[11] Wildeman C (2012). Despair by association? The mental health of mothers with children by recently incarcerated fathers. Am Sociol Rev.

[12] Green KE, Ensminger ME, Robertson JA, Juon H-S (2006). Impact of adult sons’ incarceration on African American mothers’ psychological distress. J Marriage Fam.

[13] Wildeman C, Andersen SH, Lee H, Karlson KB (2014). Parental incarceration and child mortality in Denmark. Am J Public Health.

[14] Thomas JC, Torrone E (2006). Incarceration as forced migration: effects on selected community health outcomes. Am J Public Health.

[15] Freudenberg N, Daniels J, Crum M, Perkins T, Richie BE (2005). Coming home from jail: the social and health consequences of community reentry for women, male adolescents, and their families and communities. Am J Public Health.

[16] Fu JJ, Herme M, Wickersham JA, Zelenev A, Althoff A, Zaller ND (2013). Understanding the revolving door: individual and structural-level predictors of recidivism among individuals with HIV leaving jail. AIDS Behav.

[17] Chalmers I, Bracken MB, Djulbegovic B, Garattini S, Grant J, Gulmezoglu AM (2014). How to increase value and reduce waste when research priorities are set. Lancet.

[18] Arksey HOML (2005). Scoping studies: towards a methodological framework. Int J Soc Res Meth.

[19] Arksey H, O'Malley L, Baldwin S, Harris J, Mason A (2002). Services to support carers of people with mental health problems. Social Policy Research Unit: University of York.

[20] Government of Canada. Corrections and Conditional Release Act. http://laws-lois.justice.gc.ca/eng/acts/C-44.6/20021231/P1TT3xt3.html. 2002. Accessed September 18 2014.

[21] Canadian Institute for Health Information. Health indicators 2012. https://secure.cihi.ca/free_products/health_indicators_2012_en.pdf. 2012. Accessed.

[22] Public Health Agency of Canada. Determinants of health: what makes Canadians healthy or unhealthy? http://www.phac-aspc.gc.ca/ph-sp/determinants/index-eng.php-determinants. 2011. Accessed September 19 2014

[23] The Cochrane Collaboration. Cochrane handbook for systematic reviews of interventions version 5.0.2. http://www.cochrane-handbook.org. 2009. Accessed November 11 2014

[24] American Psychiatric Association (2013). Diagnostic and statistical manual of mental disorders.

[25] Wong S (1995). Recidivism and criminal career profiles of psychopaths: a longitudinal study. Issues Forensic Psychol.

[26] Boyce J. Adult criminal court statistics in Canada, 2011/2012. http://www.statcan.gc.ca/pub/85-002-x/2013001/article/11804-eng.htm?fpv=2693-a8. 2013. Accessed November 17 2014.

[27] Kouyoumdjian FG, McIsaac KE, Liauw J, Green S, Karachiwalla F, Siu W (2015). A systematic review of randomized controlled trials of interventions to improve the health of persons during imprisonment and in the year after release. Am J Public Health.

[28] National Criminal Justice Reference Service. National criminal justice reference service: grants and funding*.* http://www.ncjrs.gov/fedgrant.html. Accessed December 14 2014.

[29] Boe R, Vuong B. Mental health trends among federal inmates. http://www.ncjrs.gov/pdffiles1/Digitization/199340-199351NCJRS.pdf. 2002. Accessed December 14 2014.

[30] Sapers H. Annual report of the correctional investigator: 2013–2014. http://www.oci-bec.gc.ca/cnt/rpt/pdf/annrpt/annrpt20132014-eng.pdf. 2014. Accessed November 25 2014.

[31] Statistics Canada. Canadian health measures survey. http://www23.statcan.gc.ca/imdb/p2SV.pl?Function=getSurvey&SDDS=5071. 2014. Accessed November 18 2014.

[32] Bureau of Justice Statistics. Data collection: survey of inmates in federal correctional facilities. http://www.bjs.gov/index.cfm?ty=dcdetail&iid=273. Accessed November 18 2014.

[33] Bureau of Justice Statistics. Data collection: survey of inmates in State correctional facilities. http://www.bjs.gov/index.cfm?ty=dcdetail&iid=275. Accessed November 18 2014.

[34] Robinson D, Mirabelli, L. Summary of findings of the 1995 CSC National Inmate Survey. http://www.csc-scc.gc.ca/research/b14e-eng.shtml. 1996. Accessed November 18 2014

[35] Johnston JC. Aboriginal offender survey: case files and interview sample, R61. http://www.csc-scc.gc.ca/research/r61e-eng.shtml. 1997. Accessed July 25 2014

[36] Zakaria D. Relationships between lifetime health risk-behaviours and self-reported human immunodeficiency virus and hepatitis C virus infection status among Canadian federal inmates. http://www.publicsafety.gc.ca/lbrr/archives/cn21491-eng.pdf. 2012. Accessed June 16 2014.

[37] Zakaria D. Relationships between health risk-behaviours, self-perceived risk for infection, and testing for human immunodeficiency virus and hepatitis C virus infections among Canadian federal inmates. http://www.csc-scc.gc.ca/research/005008-0254-eng.shtml. 2011. Accessed June 16 2014.

[38] Thompson J, Zakaria D, Grant B. Summary of the 2007 National Inmate Infectious Diseases and Risk-Behaviours Survey for Women. http://www.csc-scc.gc.ca/research/005008-0238-eng.shtml. 2011. Accessed June 16 2014.

[39] Zakaria D, Thompson JM, Jarvis A, Borgatta F. Summary of emerging findings from the 2007 National Inmate Infectious Diseases and Risk-Behaviours Survey. http://www.csc-scc.gc.ca/005/008/092/005008-0211-01-eng.pdf. 2010. Accessed June 16 2014.

[40] Zakaria D, Thompson J, Jarvis A, Smith J. Testing and treatment for human immunodeficiency virus and hepatitis C virus infections among Canadian federal inmates, R-223. http://www.csc-scc.gc.ca/research/005008-0223-eng.shtml. 2010. Accessed June 16 2014.

[41] Rezansoff SN, Moniruzzaman A, Gress C, Somers JM (2013). Psychiatric diagnoses and multiyear criminal recidivism in a Canadian provincial offender population. Psychol Public Policy Law.

[42] Martin RE, Hislop TG, Moravan V, Grams GD, Calam B (2008). Three-year follow-up study of women who participated in a cervical cancer screening intervention while in prison. Can J Public Health.

[43] Martin RE, Murphy K, Chan R, Ramsden VR, Granger-Brown A, Macaulay AC (2009). Primary health care: applying the principles within a community-based participatory health research project that began in a Canadian women’s prison. Glob Health Promot.

