# Supplementary material for: Research on the health of people who experience detention or incarceration in Canada: a scoping review
Source: BMC Public Health. 2015 Apr 25;15:419. doi: 10.1186/s12889-015-1758-6 (PMC4443600; doi:10.1186/s12889-015-1758-6)
Supplement: Additional file 1: — Search strategy and reference list for identified studies. [file 12889_2015_1758_MOESM1_ESM.docx]

Additional file 1. Search strategy

| Database(s) | Search terms | |
| --- | --- | --- |
| Medline | 1 | Prisoners/ |
|  | 2 | exp Criminals/ |
|  | 3 | Prisons/ |
|  | 4 | (correctional system or felon* or imprison* or incarcerat* or jail* or offender* or prison* or convict* or inmate*).tw. |
|  | 5 | (after prison or after parole or community reentry or ex-convict* or ex-inmate* or ex-offender* or ex-prisoner* or former convict* or former inmate* or former offender* or former prisoner* or formerly incarcerated or offender* reenter* or offender* reentry or offender* reintegrat* or offender* release or out of jail or parole* or postincarceration or post-incarceration or postprison or post-prison or postrelease or post-release or prison to community or prison to society or prisoner* reenter* or prisoner* reentry or prisoner* reintegrat* or prisoner* release* or probation* or rearrest* or recidivis* or recividate* or reconvict* or re-imprison* or re-incarcerat* or release* from prison or re-offend* or return to communit* or reoffend*).tw. |
|  | 6 | exp Juvenile Delinquency/ or "juvenile delinquent".mp. |
|  | 7 | or/1-6 |
|  | 8 | "randomized controlled trial".pt. |
|  | 9 | (random$ or placebo$ or single blind$ or double blind$ or triple blind$).ti,ab. |
|  | 10 | (retraction of publication or retracted publication).pt. |
|  | 11 | 8 or 9 or 10 |
|  | 12 | (animals not humans).sh. |
|  | 13 | ((comment or editorial or meta-analysis or practice-guideline or review or letter or journal correspondence) not "randomized controlled trial").pt. |
|  | 14 | (random sampl$ or random digit$ or random effect$ or random survey or random regression).ti,ab. not "randomized controlled trial".pt. |
|  | 15 | 11 not (12 or 13 or 14) |
|  | 16 | exp cohort studies/ |
|  | 17 | cohort$.tw. |
|  | 18 | controlled clinical trial.pt. |
|  | 19 | epidemiologic methods/ |
|  | 20 | limit 19 to yr=1966-1989 |
|  | 21 | exp case-control studies/ |
|  | 22 | (case$ and control$).tw. |
|  | 23 | cross-sectional study.mp. or exp Cross-Sectional Studies/ |
|  | 24 | questionnaire.mp. or exp Questionnaires/ |
|  | 25 | exp Health Surveys/ or exp Health Care Surveys/ or survey*.mp. |
|  | 26 | prevalence.mp. or exp Prevalence/ |
|  | 27 | risk factors.mp. or exp Risk Factors/ |
|  | 28 | or/15-18,20-27 |
|  | 29 | exp canada/ |
|  | 30 | canada.cp. |
|  | 31 | (canada or canadian$ or alberta or british columbia or columbie britannique).af. |
|  | 32 | (nova scotia or nouvelle ecosse or prince edward island or ile du prince edward or newfoundland or terre neuve or labrador or nun?v?t or nun?v?t or nwt or territoires du nord ouest or northwest territories or yukon).af. |
|  | 33 | (saskatchewan or manitoba or ontario or quebec or new brunswick or nouveau brunswick).af. |
|  | 34 | or/29-33 |
|  | 35 | 7 and 28 and 34 |
|  | 36 | remove duplicates from 35 |
|  | 37 | limit 36 to yr="1993 -Current" |
| Embase | 1 | exp prison/ or prisoner/ |
|  | 2 | criminal.mp. |
|  | 3 | exp offender/ |
|  | 4 | ex-offender.mp. |
|  | 5 | recidivism.mp. or exp recidivism/ |
|  | 6 | (correctional system or felon* or imprison* or incarcerat* or jail* or offender* or prison* or convict* or inmate*).tw. |
|  | 7 | 1 or 2 or 3 or 4 or 5 or 6 |
|  | 8 | (random$ or placebo$ or single blind$ or double blind$ or triple blind$).ti,ab. |
|  | 9 | RETRACTED ARTICLE/ |
|  | 10 | or/8-9 |
|  | 11 | (animal$ not human$).sh,hw. |
|  | 12 | (book or conference paper or editorial or letter or review).pt. not exp randomized controlled trial/ |
|  | 13 | (random sampl$ or random digit$ or random effect$ or random survey or random regression).ti,ab. not exp randomized controlled trial/ |
|  | 14 | 10 not (11 or 12 or 13) |
|  | 15 | exp cohort analysis/ |
|  | 16 | exp longitudinal study/ |
|  | 17 | exp prospective study/ |
|  | 18 | exp follow up/ |
|  | 19 | cohort$.tw. |
|  | 20 | exp case control study/ |
|  | 21 | (case$ and control$).tw. |
|  | 22 | cross-sectional study.mp. or exp cross-sectional study/ |
|  | 23 | exp prevalence/ or prevalence.mp. |
|  | 24 | risk factor.mp. or exp risk factor/ |
|  | 25 | exp health survey/ or exp health care survey/ or survey*.mp. |
|  | 26 | exp questionnaire/ or questionnaire.mp. |
|  | 27 | or/14-26 |
|  | 28 | exp canada/ |
|  | 29 | (canada or canadian$ or alberta or british columbia or columbie britannique).cp,in,ti,jw,ab. |
|  | 30 | (saskatchewan or manitoba or ontario or quebec or new brunswick or nouveau brunswick).cp,in,ti,jw,ab. |
|  | 31 | (nova scotia or nouvelle ecosse or prince edward island or ile du prince edward or newfoundland or terre neuve or labrador or nun?v?t or nun?v?t or nwt or territoires du nord ouest or northwest territories or yukon).cp,in,ti,jw,ab. |
|  | 32 | or/28-31 |
|  | 33 | 7 and 27 and 32 |
|  | 34 | limit 33 to yr="1993 -Current" |
|  | 35 | remove duplicates from 34 |
| PsycInfo | 1 | (correctional system or felon* or imprison* or incarcerat* or jail* or offender* or prison* or convict* or inmate*).tw. |
|  | 2 | exp Prisons/ or exp Prisoners/ or exp Recidivism/ or exp Criminal Rehabilitation/ or ex-offender.mp. or exp Criminals/ or exp Correctional Institutions/ or exp Incarceration/ or exp Parole/ or Juvenile Delinquency/ or Criminal Conviction/ or Criminal Record/ |
|  | 3 | 1 or 2 |
|  | 4 | (random* trial* or clinical trial* or control* trial*).mp. |
|  | 5 | exp Clinical Trials/ |
|  | 6 | exp Cohort Analysis/ or cohort study.mp. |
|  | 7 | (case control* or case-control*).mp. |
|  | 8 | longitudinal study.mp. or exp Longitudinal Studies/ |
|  | 9 | exp Prospective Studies/ or prospective study.mp. |
|  | 10 | exp Followup Studies/ |
|  | 11 | cross-sectional study.mp. |
|  | 12 | risk factor.mp. or exp Risk Factors/ |
|  | 13 | prevalence.mp. |
|  | 14 | exp Surveys/ or survey*.mp. or exp Epidemiology/ |
|  | 15 | questionnaire.mp. or exp Questionnaires/ |
|  | 16 | or/4-15 |
|  | 17 | (canada or canadian$ or alberta or british columbia or columbie britannique).mp,in,cp,pl,jx,jw,jn,so. |
|  | 18 | (saskatchewan or manitoba or ontario or quebec or new brunswick or nouveau brunswick).mp,in,cp,pl,jx,jw,jn,so. |
|  | 19 | (nova scotia or nouvelle ecosse or prince edward island or ile du prince edward or newfoundland or labrador or nun?v?t or nwt or northwest territories or territoires du nord ouest or yukon).mp,in,cp,pl,jx,jw,jn,so. |
|  | 20 | or/17-19 |
|  | 21 | 3 and 16 and 20 |
|  | 22 | limit 21 to yr="1993 -Current" |
|  | 23 | remove duplicates from 22 |
| EBSCO through UofT Popular Databases Ebscohost: Social Sciences Abstracts (1983-present), CINAHL (1981-present), Criminal Justice Abstracts (?) | ((prison* or jail* or inmate* or offender*))  AND ((canad*) or (ontario) or ("british columbia") or (saskatchewan) or (manitoba) or (quebec) or ("new brunswick") or ("nouveau brunswick") or ("nova scotia") or (“nouvelle ecosse”) or ("prince edward island") or (“ile du prince edouard”) or (newfoundland) or (“terre neuve”) or (labrador) or (nunavut) or ("nwt") or ("northwest territories") or (“territoires du nord ouest”) or (yukon))  AND (("random* trial") OR ("clinical trial*") OR ("control* trial*") OR (cohort) OR (“case-control*”) OR (“case control*”) OR (“cross-section*”))  Limiters: Published Date: 19930101-20141231 | |
| Proquest: Eric (1966-), Proquest Criminal Justice, Proquest Dissertations and Theses Full Text (no start date), Proquest Dissertations and Theses: UK and Ireland, Social Services Abstracts (1979-present), Sociological Abstracts (1952-current) | (ti("random* trial") OR ti(cohort*) OR ti(cross-section*) OR ti(case-control*) OR ti(prospective) OR ti("controlled trial") OR ti("RCT") OR ab("random* trial") OR ab(cohort*) OR ab(cross-section*) OR ab(case-control*) OR ab(prospective) OR ab("controlled trial") OR ab("RCT") OR ti("clinical trial") OR ab("clinical trial"))  AND ti(prison* OR jail OR inmate) AND ab(prison* OR jail OR inmate)  AND (ti(canada* OR ontario OR ("british columbia") OR ("colombie britannique") OR saskatchewan OR manitoba OR quebec OR ("new brunswick") OR ("nouveau brunswick") OR ("nova scotia") OR ("nouvelle ecosse") OR ("prince edward island") OR ("ile du prince edouard") OR newfoundland OR ("terre neuve") OR labrador OR nunavut OR ("nwt") OR ("territoires du nord ouest") OR ("northwest territories") OR (yukon)) OR ab(canada* OR ontario OR ("british columbia") OR ("colombie britannique") OR saskatchewan OR manitoba OR quebec OR ("new brunswick") OR ("nouveau brunswick") OR ("nova scotia") OR ("nouvelle ecosse") OR ("prince edward island") OR ("ile du prince edouard") OR newfoundland OR ("terre neuve") OR labrador OR nunavut OR ("nwt") OR ("territoires du nord ouest") OR ("northwest territories") OR (yukon))) | |
| Cochrane Library | Title/abstract/keywords: (prison* or jail* or inmate* or offender*) and (canad*) | |
| Web of Science | (Title= prison and Title=Canada and Topic=study design)  TI=(prison* or jail* or inmate* or offender*)  AND TI=((canad*) or (ontario) or ("british columbia") or (saskatchewan) or (manitoba) or (quebec) or ("new brunswick") or ("nouveau brunswick") or ("nova scotia") or (nouvelle ecosse) or ("prince edward island") or (ile du prince edouard) or (newfoundland) or (terre neuve) or (labrador) or (nunavut) or ("nwt") or ("northwest territories") or (territoires du nord ouest) or (yukon))  AND TS=(("random* trial*") OR ("clinical trial*") OR ("control* trial*") OR (cohort) OR (“case-control*”) OR (“case control*”) OR (“cross-section*”)) | |
| Scopus | (TITLE-ABS-KEY((**prison*** OR **jail*** OR **inmate*** OR **offender***)) AND TITLE-ABS-KEY((**"random* trial*"**) OR (**"clinical trial*"**) OR (**"control* trial*"**) OR (**cohort**) OR (**"case-control*"**) OR (**"case control*"**) OR (**"cross-section*"**)) AND TITLE-ABS-KEY((**canad***) OR (**ontario**) OR (**"british columbia"**) OR (**saskatchewan**) OR (**manitoba**) OR (**quebec**) OR (**"new brunswick"**) OR (**"nouveau brunswick"**) OR (**"nova scotia"**) OR (**"nouvelle ecosse"**) OR (**"prince edward island"**) OR (**"ile du prince edouard"**) OR (**newfoundland**) OR (**"terre neuve"**) OR (**labrador**) OR (**nunavut**) OR (**"nwt"**) OR (**"northwest territories"**) OR (**"territoires du nord ouest"**) OR **yukon**)) AND PUBYEAR > **1992** | |

Additional file 1. Reference list for 194 studies conducted from 1993 to 2014 on the health status of people who have experienced detention in Canada [[1-219](#_ENREF_1)]

1. Abracen J, Looman J, Anderson D. **Alcohol and drug abuse in sexual and nonsexual violent offenders**. *Sex Abuse* 2000, **12**(4):263-274.

2. Abracen J, Looman J, Di Fazio R, Kelly T, Stirpe T. **Patterns of attachment and alcohol abuse in sexual and violent non-sexual offenders**. *Journal of Sexual Aggression* 2006, **12**(1):19-30.

3. Abracen J, Mailloux DL, Serin RC, Cousineau C, Malcom PB, Looman J. **A model for the assessment of static and dynamic factors in sexual offenders**. *Journal of sex research* 2004, **41**(4):321-328.

4. Ahmed AG, Lepnurm M. **Seclusion practice in a Canadian forensic psychiatric hospital**. *Journal of the American Academy of Psychiatry & the Law* 2001, **29**(3):303-309.

5. Alary M, Godin G, Lambert G. **Étude de prévalence du VIH et de l’hépatite C chez les personnes incarcérées au Québec et pistes pour l’intervention.** <http://www.msss.gouv.qc.ca/sujets/prob_sante/itss/download.php%3Ff=63c832872bd9bbaae6bee20076203959>. 2005. Accessed December 1 2014.

6. Poulin C, Alary M, Lambert G, Godin G, Landry S, Gagnon H, Demers E, Morarescu E, Rochefort J, Claessens C. **Prevalence of HIV and hepatitis C virus infections among inmates of Quebec provincial prisons**. *Canadian Medical Association journal* 2007, **177**(3):252-256.

7. Allenby K, Taylor K, Cosette M, Fortin D. **A profile of women who sexually offend.** <http://www.csc-scc.gc.ca/research/005008-0274-eng.shtml>. 2012. Accessed July 29 2014.

8. Archambault K SL, Wilton G, & Cousineau C. **Initial results of the Computerized Mental Health Intake Screening System (CoMHISS) for Federally Sentenced Women. .** <http://www.csc-scc.gc.ca/research/005008-0230-eng.shtml>. 2010. Accessed.

9. Barrett M, Allenby K, Taylor K. **Twenty years later: Revisiting The Task Force on Federally Sentenced Women.** <http://www.csc-scc.gc.ca/research/005008-0222-eng.shtml>. 2010. Accessed July 24, 2014.

10. Bartlett L, Kanellos Sutton, M, van Wylick, R. **Immunization rates in a Canadian juvenile corrections facility**. *Journal of Adolescent Health Care* 2008, **43**(6):609-611.

11. Beaudette J. **Prevalence of Mental Health Disorders Among Incoming Federal Offenders: Atlantic, Ontario, & Pacific Regions.** <http://www.csc-scc.gc.ca/research/005008-err13-3-eng.shtml>. 2013. Accessed July 2, 2014.

12. Beauregard E, Stone MR, Proulx J, Michaud P. **Sexual murderers of children: Developmental, precrime, crime, and postcrime factors**. *International Journal of Offender Therapy and Comparative Criminology* 2008, **52**(3):253-269.

13. Bell A, Trevethan S, Allegri N. **A Needs Assessment of Federal Aboriginal Women Offenders.** [http://www.csc-scc.gc.ca/research/r156-eng.shtml - 8](http://www.csc-scc.gc.ca/research/r156-eng.shtml#8). 2004. Accessed July 25, 2014.

14. Bell A, Flight J. **An Evaluation of the Spirit of a Warrior Program for Women Offenders.** <http://www.csc-scc.gc.ca/research/r180-eng.shtml>. 2006. Accessed December 1, 2014.

15. Bezeau SC. **Neuropsychological correlates of youth psychopathy**. *Dissertation Abstracts International* 2004, **66**(5-B):2809.

16. Bistodeau D, Daigle M. **Suicide prevention in a prison setting: Assessment of an innovative experience**. *Revue de Psychoeducation, Revue de Psychoeducation et d'Orientation* 2000, **29**(1):49-64.

17. Blanchette K, Eljdupovic-Guzina G. **Results of a Pilot Study of the Peer Support Program for Women Offenders.** [http://www.csc-scc.gc.ca/research/r73e-eng.shtml - Toc419531818](http://www.csc-scc.gc.ca/research/r73e-eng.shtml#Toc419531818). 1998. Accessed July 28, 2014.

18. Blanchette K, Flight, J, Verbrugge, P, Gobeil, R, Taylor, K. **Dialectical Behaviour Therapy within a Women's Structured Living Environment.** <http://www.csc-scc.gc.ca/research/005008-0241-eng.shtml>. 2011. Accessed July 25, 2014.

19. Bland RC, Newman SC, Thompson AH, Dyck RJ. **Psychiatric disorders in the population and in prisoners**. *International journal of law and psychiatry* 1998, **21**(3):273-279.

20. Boe R, Vuong B. **Mental health trends among federal inmates.** https://<http://www.ncjrs.gov/pdffiles1/Digitization/199340-199351NCJRS.pdf>. 2002. Accessed December 14 2014.

21. Bonnycastle KD, Villebrun C. **Injecting Risk Into Prison Sentences: A Quantitative Analysis of a Prisoner-Driven Survey to Measure HCV/HIV Seroprevalence, Risk Practices, and Viral Testing at One Canadian Male Federal Prison**. *Prison Journal* 2011, **91**(3):325-346.

22. Brochu S, Cousineau M-M, Gillet M, Cournoyer L-G, Pernanen K, Motiuk L. **Drugs, alcohol, and criminal behaviour: A profile of inmates in Canadian federal institutions**. *Forum on Corrections Research* 2001, **13**(3):20-24.

23. Brochu S, Guyon L, Desjardins L. **Comparative profiles of addicted adult populations in rehabilitation and correctional services**. *Journal of substance abuse treatment* 1999, **16**(2):173-182.

24. Brown SL, Forth AE. **Psychopathy and sexual assault: static risk factors, emotional precursors, and rapist subtypes**. *Journal of Consulting & Clinical Psychology* 1997, **65**(5):848-857.

25. Brunelle C, Douglas RL, Pihl RO, Stewart SH. **Personality and substance use disorders in female offenders: A matched controlled study**. *Personality and Individual Differences* 2009, **46**(4):472-476.

26. Burchell AN, Calzavara LM, Myers T, Schlossberg J, Millson M, Escobar M, Wallace E, Major C. **Voluntary HIV testing among inmates: sociodemographic, behavioral risk, and attitudinal correlates**. *Journal of Acquired Immune Deficiency Syndromes: JAIDS* 2003, **32**(5):534-541.

27. Calzavara L, Burchell A. **Developing effective HIV prevention programs for inmates: results from an Ontario-wide survey**. *Canadian HIV-AIDS Policy &amp; Law Newsletter* 1999, **5**(1):32-34.

28. Calzavara L, Ramuscak N, Burchell AN, Swantee C, Myers T, Ford P, Fearon M, Raymond S. **Prevalence of HIV and hepatitis C virus infections among inmates of Ontario remand facilities**. *Canadian Medical Association journal* 2007, **177**(3):257-261.

29. Calzavara LM, Burchell AN, Schlossberg J, Myers T, Escobar M, Wallace E, Major C, Strike C, Millson M. **Prior opiate injection and incarceration history predict injection drug use among inmates**. *Addiction* 2003, **98**(9):1257-1265.

30. Calzavara LM, Major C, Myers T, Schlossberg J, Millson M, Wallace E, Rankin J, Fearon M. **The prevalence of HIV-1 infection among inmates in Ontario, Canada**. *Canadian Journal of Public HealthRevue Canadienne de Sante Publique* 1995, **86**(5):335-339.

31. Calzavara LM, Major C, Myers T, Schlossberg J, Millson M, Wallace E, Rankin J, Fearon M. **Reducing volunteer bias: using left-over specimens to estimate rates of HIV infection among inmates in Ontario, Canada**. *AIDS* 1995, **9**(6):631-637.

32. Campbell MA. **The relative contribution of psychopathy and traditional risk factors in predicting young offender recidivism**. *Dissertation Abstracts International* 2003, **65**(3-B):1539.

33. Campbell MA, Porter S, Santor D. **Psychopathic traits in adolescent offenders: An evaluation of criminal history, clinical, and psychosocial correlates**. *Behavioral sciences &amp; the law* 2004, **22**(1):23-47.

34. Canadian HIV/AIDS Legal Network. **Canada: study provides further evidence of risk of hepatitis C and HIV transmission in prisons**. *HIV/AIDS Policy &amp; Law Review / Canadian HIV/AIDS Legal Network* 2004, **9**(3):45-46.

35. Cesaroni C, Peterson-Badali M. **Young Offenders in Custody: Risk and Adjustment**. *Criminal Justice and Behavior* 2005, **32**(3):251-277.

36. Chubaty DE. **Victimization, fear, and coping in prison**. *Dissertation Abstracts International* 2001, **62**(2-B):1071.

37. **A health care needs assessment of federal inmates in Canada**. *Canadian journal of public health = Revue canadienne de sante publique* 2004, **95 Suppl 1**:S9-63.

38. Colantonio A, Kim H, Allen S, Asbridge M, Petgrave J, Brochu S. **Traumatic Brain Injury and Early Life Experiences Among Men and Women in a Prison Population**. *Journal of correctional health care : the official journal of the National Commission on Correctional Health Care* 2014.

39. Cooper BS, Herve H, Yuille JC. **Psychopathy and memory for violence**. *The International Journal of Forensic Mental Health* 2007, **6**(2):123-135.

40. Crocker A, Cote G, Farthing D, Daigle M, Toupin J. **Rates and correlates of intellectual disability in a Canadian prison**. *14th World Congress of the International Association for the Scientific Study of Disabilities, IASSID 2012 Halifax, NS Canada* 2012, **56**(7-8):682.

41. Crocker AG, Cote G, Toupin J, St-Onge B. **Rate and characteristics of men with an intellectual disability in pre-trial detention**. *Journal of Intellectual &amp; Developmental Disability* 2007, **32**(2):143-152.

42. Grant B, Furlong, A, Hume, L, White, T, Doherty, S. **The Women Offender Substance Abuse Programming: Interim Research Report.** <http://www.csc-scc.gc.ca/research/r171-eng.shtml>. 2008. Accessed July 28. 2014.

43. Correctional Service Canada. **Tuberculosis Prevention and Control in Canadian Federal Prisons 1998: Reported Results of the Correctional Service of Canada Tuberculosis Tracking System.** <http://publications.gc.ca/collections/Collection/JS82-94-1998E.pdf>. 2000. Accessed December 2 2014.

44. Correctional Service Canada. **Infectious Diseases Prevention and Control in Canadian Federal Penitentiaries 2000-01.** <http://publications.gc.ca/collections/Collection/JS82-104-2001E.pdf>. 2003. Accessed November 18, 2014.

45. Correctional Service Canada. **Infectious Disease Surveillance in Canadian Federal Penitentiaries 2007-2008.** <http://www.csc-scc.gc.ca/text/pblct/infdscfp-2007-08/index-eng.shtml>. 2008. Accessed December 15, 2014.

46. Daigle M, Côté G. **Dépistage systématique et prise en charge des hommes incarcérés suicidaires.** <http://www.crise.ca/e-docs/daigle2002_hommes.pdf>. 2002. Accessed December 2 2014.

47. Daigle M. **MMPI inmate profiles: suicide completers, suicide attempters, and non-suicidal controls**. *Behavioral sciences &amp; the law* 2004, **22**(6):833-842.

48. Daigle MS, Cote G. **Nonfatal suicide-related behavior among inmates: testing for gender and type differences**. *Suicide & life-threatening behavior* 2006, **36**(6):670-681.

49. Daigle MS, Labelle R, Cote G. **Further evidence of the validity of the Suicide Risk Assessment Scale for prisoners**. *International Journal of Law & Psychiatry* 2006, **29**(5):343-354.

50. Day DM, Hart TA, Wanklyn SG, McCay E, Macpherson A, Burnier N. **Potential mediators between child abuse and both violence and victimization in juvenile offenders**. *Psychological Services* 2013, **10**(1):1-11.

51. De P, Connor N, Bouchard F, Sutherland D. **HIV and hepatitis C virus testing and seropositivity rates in Canadian federal penitentiaries: A critical opportunity for care and prevention**. *Canadian Journal of Infectious Diseases* 2004, **15**(4):221-225.

52. DeBeck K, Kerr T, Li K, Milloy MJ, Montaner J, Wood E. **Incarceration and drug use patterns among a cohort of injection drug users**. *Addiction* 2009, **104**(1):69-76.

53. Buxton JA, Rothon D, Durigon M, Lem M, Tu AW, Remple VP, Cook D, Krajden M. **Hepatitis C and HIV prevalence using oral mucosal transudate, and reported drug use and sexual behaviours of youth in custody in British Columbia**. *Canadian Journal of Public HealthRevue Canadienne de Sante Publique* 2009, **100**(2):121-124.

54. Derkzen D, Booth, L, McConnell, A, & Taylor, K. **Mental health needs of federal women offenders. .** <http://www.csc-scc.gc.ca/research/005008-0267-eng.shtml>. 2012. Accessed July 2, 2014.

55. Derkzen DM, Allenby, K. **Assessment of the Aboriginal Women’s Maintenance Program.** <http://www.csc-scc.gc.ca/research/005008-b51-eng.shtml>. 2012. Accessed July 28. 2014.

56. Dietrich A. **Childhood maltreatment and revictimization: The role of affect dysregulation, interpersonal relatedness difficulties and posttraumatic stress disorder**. *Journal of Trauma & Dissociation* 2007, **8**(4):25-51.

57. Douglas KS, Guy LS, Edens JF, Boer DP, Hamilton J. **The Personality Assessment Inventory as a proxy for the Psychopathy Checklist Revised: testing the incremental validity and cross-sample robustness of the Antisocial Features Scale**. *Assessment* 2007, **14**(3):255-269.

58. Dowden C, Blanchette K. **An investigation into the Characteristics of Substance-Abusing Women Offenders: Risk, Need, and Post-release outcome.** <http://www.csc-scc.gc.ca/research/092/r81_e.pdf>. 1999. Accessed December 2, 2014.

59. Dufour A, Alary M, Poulin C, Allard F, Noel L, Trottier C, Lepine D, Hankins C. **Prevalence and risk behaviours for HIV infection among inmates of a provincial prison in Quebec City**. *AIDS* 1996, **10**(9):1009-1015.

60. Elgar FJ, Knight J, Worrall GJ, Sherman G. **Behavioural and substance use problems in rural and urban delinquent youths**. *Canadian Journal of Psychiatry - Revue Canadienne de Psychiatrie* 2003, **48**(9):633-636.

61. Farley J, Truong A, Horvath G, Nguyen T, Shum W. **Re-infection of hepatitis C virus infection in HIV/HCV co-infected inmates of correctional institutions, Canada**. *11th International Congress on Drug Therapy in HIV Infection Glasgow United Kingdom* 2012, **15**:87.

62. Farley J, Truong A, Nguyen T, Shum W. **Ten year follow up of treatment of hepatitis C in intravenous drug users**. *15th International Congress on Infectious Diseases, ICID 2012 Bangkok Thailand* 2012, **16**:e49.

63. Farley J, Vasdev S, Fischer B, Haydon E, Rehm J, Farley TA. **Feasibility and outcome of HCV treatment in a Canadian federal prison population**. *American Journal of Public Health* 2005, **95**(10):1737-1739.

64. Farley JD, Wong VK, Chung HV, Lim E, Walters G, Farley TA, Yoshida EM. **Treatment of chronic hepatitis C in Canadian prison inmates**. *Canadian Journal of Gastroenterology* 2005, **19**(3):153-156.

65. Farrell MacDonald S, Forrester P, Trainor E, Varis D. **The Cultural, Social and Substance Use Histories of Male Offenders Enrolled in the Aboriginal Offender Substance Abuse Program (AOSAP).** <http://www.csc-scc.gc.ca/research/005008-rs13-02-eng.shtml>. 2013. Accessed July 24, 2014.

66. Farrell S, Ross J, Ternes M, Kunic D. **Prevalence of Injection Drug Use among Male Offenders.** <http://www.csc-scc.gc.ca/research/005008-rs10-02-eng.shtml>. 2010. Accessed July 29, 2014.

67. Finlay J. **Keeping kids safe in custody: Youths' perceptions of safety while incarcerated in Canada**. *Dissertation Abstracts International* 2009, **70**(12-A):4864.

68. Ford PM, Alifo A, Connop PJ, Panaro L, Zoutman D. **Seroprevalence of HIV-1 in a male medium security penitentiary--Ontario**. *Canada communicable disease report = Releve des maladies transmissibles au Canada* 1994, **20**(6):45-47.

69. Ford PM, Pearson M, Sankar-Mistry P, Stevenson T, Bell D, Austin J. **HIV, hepatitis C and risk behaviour in a Canadian medium-security federal penitentiary. Queen's University HIV Prison Study Group**. *Qjm* 2000, **93**(2):113-119.

70. Ford PM, White C, Kaufmann H, MacTavish J, Pearson M, Ford S, Mistry PS, Connop P. **Seroprevalence of hepatitis C in a Canadian federal penitentiary for women**. *Canada communicable disease report = Releve des maladies transmissibles au Canada* 1995, **21**(14):132-134.

71. Ford PM, White C, Kaufmann H, MacTavish J, Pearson M, Ford S, Sankar-Mistry P, Connop P. **Voluntary anonymous linked study of the prevalence of HIV infection and hepatitis C among inmates in a Canadian federal penitentiary for women**. *CMAJ Canadian Medical Association Journal* 1995, **153**(11):1605-1609.

72. Gabor T. **Deaths in Custody: Final Report.** <http://www.oci-bec.gc.ca/cnt/rpt/oth-aut/oth-aut20070228-eng.aspx>. 2007. Accessed September 22, 2014.

73. Gagnon H, Godin G, Alary M, Lambert G, Lambert LD, Landry S. **Prison inmates' intention to demand that bleach be used for cleaning tattooing and piercing equipment**. *Canadian Journal of Public HealthRevue Canadienne de Sante Publique* 2007, **98**(4):297-300.

74. Gander S, Scholten V, Osswald I, Sutton M, van Wylick R. **Cervical dysplasia and associated risk factors in a juvenile detainee population**. *Journal of Pediatric &amp; Adolescent Gynecology* 2009, **22**(6):351-355.

75. Pearson M, Mistry PS, Ford PM. **Voluntary screening for hepatitis C in a Canadian federal penitentiary for men**. *Can Commun Dis Rep* 1995, **21**(14):134-136.

76. Gerber GJ, Prince PN, Duffy S, McDougall L, Cooper J, Dowler S. **Adjustment, integration, and quality of life among forensic patients receiving community outreach services**. *The International Journal of Forensic Mental Health* 2003, **2**(2):129-136.

77. Ginsburg JID. **Using motivational interviewing to enhance treatment readiness in offenders with symptoms of alcohol dependence**. *Dissertation Abstracts International* 2000, **61**(8-B):4404.

78. Goldberg E, Millson P, Rivers S, Manning SJ, Leslie K, Read S, Shipley C, Victor JC. **A human immunodeficiency virus risk reduction intervention for incarcerated youth: A randomized controlled trial**. *Journal of Adolescent Health Care* 2009, **44**(2):136-145.

79. Gordon A. **Self-Injury Incidents in Correctional Service of Canada Institutions Over a Thirty-Month Period.** <http://www.csc-scc.gc.ca/research/005008-0233-01-eng.shtml>. 2010. Accessed June 16, 2014.

80. Grant B, Gal M. **Case managment preparation for release and day parole outcome.** <http://www.csc-scc.gc.ca/research/092/r63_e.pdf>. 1998. Accessed July 31, 2014.

81. Grant B, Kunic D, MacPherson P, McKeown C, Hansen E. **The High Intensity Substance Abuse Program (HISAP): Results from the Pilot Programs.** <http://www.csc-scc.gc.ca/research/r140-eng.shtml>. 2004. Accessed July 28, 2014.

82. Grant B, Varis DD, Lefebvre D. **Intensive Support Units (ISU) for Federal Offenders with Substance Abuse Problems: An Impact Analysis.** <http://www.csc-scc.gc.ca/research/r151-eng.shtml>. 2005. Accessed July 30. 2014.

83. Gretton HM, Clift RJW. **The mental health needs of incarcerated youth in British Columbia, Canada**. *International journal of law and psychiatry* 2011, **34**(2):109-115.

84. Hopley AA, Brunelle C. **Personality mediators of psychopathy and substance dependence in male offenders**. *Addictive Behaviors* 2012, **37**(8):947-955.

85. Horn M, Potvin S, Allaire JF, Cote G, Gobbi G, Benkirane K, Vachon J, Dumais A. **Male inmate profiles and their biological correlates**. *Canadian journal of psychiatry Revue canadienne de psychiatrie* 2014, **59**(8):441-449.

86. The John Howard Society of Toronto. **Homeless and jailed: Jailed and homeless.** [http://www.johnhoward.ca/document/JHS-Toronto Report Homeless and Jailed.pdf](http://www.johnhoward.ca/document/JHS-Toronto%20Report%20Homeless%20and%20Jailed.pdf). 2010. Accessed July 31 2014.

87. Johnson S, Cheverie M, Moser A. **Assessing the impact of enhanced drug interdiction activities at Kington Penitentiary: A pilot study.** <http://www.csc-scc.gc.ca/research/005008-0232-eng.shtml>. 2010. Accessed July 28. 2014.

88. Johnson S, MacDonald SF, Cheverie M. **Characteristics of participants in the methadone maintenance treatment (MMT) program.** <http://www.csc-scc.gc.ca/research/005008-0253-eng.shtml>. 2011. Accessed December 4, 2014.

89. Wong SC, Gordon A, Gu D. **Assessment and treatment of violence-prone forensic clients: an integrated approach**. *British Journal of Psychiatry - Supplementum* 2007, **49**:s66-74.

90. Johnson S, MacDonald SF, Cheverie M, Myrick C, Fischer B. **Prevalence and trends of non-medical opioid and other drug use histories among federal correctional inmates in methadone maintenance treatment in Canada**. *Drug and alcohol dependence* 2012, **124**(1-2):172-176.

91. Johnston JC. **Northern Aboriginal Offenders in Federal Custody: A Profile.** <http://www.csc-scc.gc.ca/research/r36e-eng.shtml>. 1994. Accessed July 25, 2014.

92. Johnston JC. **Aboriginal Offender Survey: Case files and interview sample, R61.** <http://www.csc-scc.gc.ca/research/r61e-eng.shtml>. 1997. Accessed July 25 2014.

93. Porter S, Woodworth M, Earle J, Drugge J, Boer D. **Characteristics of sexual homicides committed by psychopathic and nonpsychopathic offenders**. *Law & Human Behavior* 2003, **27**(5):459-470.

94. Wood E, Li K, Small W, Montaner JS, Schechter WT, Kerr T. **Recent incarceration independently associated with syringe sharing by injection drug users**. *Public health reports* 2005, **120**(2):150-156.

95. Woodworth M, Porter S. **In cold blood: characteristics of criminal homicides as a function of psychopathy**. *J Abnorm Psychol* 2002, **111**(3):436-445.

96. Kinner S, Milloy MJ, Wood E, Qi J, Zhang R, Kerr T. **Incidence and risk factors for non-fatal overdose among a cohort of recently incarcerated illicit drug users**. *Addictive Behaviors* 2012, **37**(6):691-696.

97. Kouyoumdjian F, Calzavara LM, Kiefer L, Main C, Bondy SJ. **Drug use prior to incarceration and associated socio-behavioural factors among males in a provincial correctional facility in Ontario, Canada**. *Canadian Journal of Public Health* 2014, **105**(3):198-202.

98. Kouyoumdjian FG, Main C, Calzavara LM, Kiefer L. **Prevalence and predictors of urethral chlamydia and gonorrhea infection in male inmates in an Ontario correctional facility**. *Canadian Journal of Public HealthRevue Canadienne de Sante Publique* 2011, **102**(3):220-224.

99. Kroner DG, Kang T, Mills JF, Harris AJR, Green MM. **Reliabilities, validities, and cutoff scores of the depression hopelessness suicide screening form among women offenders**. *Criminal Justice and Behavior* 2011, **38**(8):779-795.

100. Kroner DG, Loza W. **Evidence for the efficacy of self-report in predicting nonviolent and violent criminal recidivism**. *Journal of Interpersonal Violence* 2001, **16**(2):168-177.

101. Kroner DG, Mills JF. **The accuracy of five risk appraisal instruments in predicting institutional misconduct and new convictions**. *Criminal Justice and Behavior* 2001, **28**(4):471-489.

102. Kunic D, Grant BA. **The Computerized Assessment of Substance Abuse (CASA):Results from the Demonstration Project.** <http://www.csc-scc.gc.ca/research/r173-eng.shtml>. 2006. Accessed July 29, 2014.

103. Lafortune D. **Prevalence and screening of mental disorders in short-term correctional facilities**. *International Journal of Law &amp; Psychiatry* 2010, **33**(2):94-100.

104. Lafortune D, Vacheret M. **[Prescription of psychotropic medication in inmates of Quebec's correctional facilities]**. *Sante mentale au Quebec* 2009, **34**(2):147-170.

105. Burke HC. **Psychopathy and treatment outcome in incarcerated violent offender program participants**. Simon Fraser University; 2002.

106. Langner N, Barton J, McDonagh D, Noël C, Bouchard F. **Rates of prescribed medication use by women in prison.** <http://www.csc-scc.gc.ca/publications/forum/e142/e142c-eng.shtml>. 2002. Accessed December 4 2014.

107. Lasnier B, Cantinotti M, Guyon L, Royer A, Brochu S, Chayer L. **Implementing an indoor smoking ban in prison: Enforcement issues and effects on tobacco use, exposure to second-hand smoke and health of inmates**. *Canadian Journal of Public Health* 2011, **102**(4):249-253.

108. Lee Z, Klaver JR, Hart SD, Moretti MM, Douglas KS. **Short-term stability of psychopathic traits in adolescent offenders**. *Journal of Clinical Child & Adolescent Psychology* 2009, **38**(5):595-605.

109. Lee Z, Vincent GM, Hart SD, Corrado RR. **The validity of the Antisocial Process Screening Device as a self-report measure of psychopathy in adolescent offenders**. *Behavioral sciences & the law* 2003, **21**(6):771-786.

110. Looman J, Abracen J. **Substance abuse among high-risk sexual offenders: Do measures of lifetime history of substance abuse add to the prediction of recidivism over actuarial risk assessment instruments?** *Journal of Interpersonal Violence* 2011, **26**(4):683-700.

111. Looman J, Abracen J. **The Static-99R: Are there really differences between the normative groups?** *International Journal of Offender Therapy and Comparative Criminology* 2012, **57**(7):888-907.

112. Looman J, Abracen J, Serin R, Marquis P. **Psychopathy, treatment change, and recidivism in high-risk, high-need sexual offenders**. *Journal of Interpersonal Violence* 2005, **20**(5):549-568.

113. Louth S, Hare RD, Linden W. **Psychopathy and alexithymia in female offenders**. *Canadian Journal of Behavioural Science* 1998, **30**(2):91-98.

114. MacPherson P, Chudley AE, Grant BA. **Fetal Alcohol Spectrum DIsorder in a correctional population: Prevalence, screening, and characteristics.** <http://www.csc-scc.gc.ca/research/005008-0247-eng.shtml>. 2011. Accessed July 24 2014.

115. MacSwain M-A, Cheverie M. **Comparing the Mental Health Treatment and Abuse Histories of Men and Women Methadone Maintenance Treatment Program (MMTP) Participants.** <http://www.csc-scc.gc.ca/research/005008-rs12-9-eng.shtml>. 2012. Accessed July 2, 2014.

116. MacSwain M-A, Cheverie M. **Comparing the Mental Health Treatment and Abuse Histories of Aboriginal and Non-Aboriginal Participants of the Methadone Maintenance Treatment Program (MMTP).** <http://www.csc-scc.gc.ca/research/005008-rs12-8-eng.shtml>. 2012. Accessed July 2, 2014.

117. MacSwain M-A, Cheverie M, Farrell MacDonald S, Johnson S. **Institutional Adjustment of Methadone Maintenance Treatment Program (MMTP) Participants: A Comparative Study.** <http://www.csc-scc.gc.ca/research/005008-err12-4-eng.shtml>. 2012. Accessed July 28, 2014.

118. Marshall LE, Marshall WL. **Sexual Addiction in Incarcerated Sexual Offenders**. *Sexual Addiction &amp; Compulsivity* 2006, **13**(4):377-390.

119. Marshall WL, Kennedy P, Yates P. **Issues concerning the reliability and validity of the diagnosis of sexual sadism applied in prison settings**. *Sexual abuse : a journal of research and treatment* 2002, **14**(4):301-311.

120. Marshall WL, Serran GA, Cortoni FA. **Childhood attachments, sexual abuse, and their relationship to adult coping in child molesters**. *Annals of Sex Research* 2000, **12**(1):17-26.

121. Martin MS, Dorken SK, Colman I, McKenzie K, Simpson AI. **The incidence and prediction of self-injury among sentenced prisoners**. *Canadian journal of psychiatry Revue canadienne de psychiatrie* 2014, **59**(5):259-267.

122. Martin RE. **A review of a prison cervical cancer screening program in British Columbia**. *Canadian Journal of Public HealthRevue Canadienne de Sante Publique* 1998, **89**(6):382-386.

123. Martin RE, Adamson S, Korchinski M, GrangerBrown A, Ramsden VR, Buxton JA, EspinozaMagana N, Pollock SL, Smith MJF, Macaulay AC *et al*. **Incarcerated women develop a nutrition and fitness program: Participatory research**. *International Journal of Prisoner Health* 2013, **9**(3):142-150.

124. Martin RE, Gold F, Murphy W, Remple V, Berkowitz J, Money D. **Drug use and risk of bloodborne infections: a survey of female prisoners in British Columbia**. *Canadian Journal of Public HealthRevue Canadienne de Sante Publique* 2005, **96**(2):97-101.

125. Martin RE, Hislop TG, Moravan V, Grams GD, Calam B. **Three-year follow-up study of women who participated in a cervical cancer screening intervention while in prison**. *Canadian Journal of Public HealthRevue Canadienne de Sante Publique* 2008, **99**(4):262-266.

126. Mela M. **Benefits of Psychotropic Drugs in High Risk/High Need Sexual Offenders.** <http://www.csc-scc.gc.ca/research/005008-ers12-02-eng.shtml>. 2012. Accessed July 2, 2014.

127. Michel S, Gobeil R, McConnell A. **Older incarcerated women offenders: Social support and health needs. .** <http://www.csc-scc.gc.ca/research/005008-0275-eng.shtml>. 2012. Accessed July 2, 2014.

128. Milloy MJS, Buxton J, Wood E, Li K, Montaner JS, Kerr T. **Elevated HIV risk behaviour among recently incarcerated injection drug users in a Canadian setting: A longitudinal analysis**. *BMC Public Health* 2009, **9**.

129. Mills JF, Green K, Reddon JR. **An evaluation of the Psychache Scale on an offender population**. *Suicide & life-threatening behavior* 2005, **35**(5):570-580.

130. Mills JF, Kroner DG. **Screening for suicide risk factors in prison inmates: Evaluating the efficiency of the Depression, Hopelessness and Suicide Screening Form (DHS)**. *Legal and Criminological Psychology* 2005, **10**(1):1-12.

131. Mills JF, Kroner DG. **The effect of discordance among violence and general recidivism risk estimates on predictive accuracy**. *Criminal Behaviour & Mental Health* 2006, **16**(3):155-166.

132. Mowat-Leger V. **Risk factors for violence: A comparison of domestic batterers and other violent and non-violent offenders**. *Dissertation Abstracts International* 2001, **63**(4-B):2046.

133. Mullins P, Farrell MacDonald, S. **Offender Substance Use Patterns – Aboriginal and Non-Aboriginal Offenders.** <http://www.csc-scc.gc.ca/research/005008-rs12-10-eng.shtml>. 2012. Accessed July 24, 2014.

134. Murphy A, Chittenden M, The McCreary Centre Society. **Time Out II: A Profile of BC Youth in Custody.** <http://www.mcs.bc.ca/pdf/time_out_2.pdf>. 2005. Accessed.

135. Nicholls TL, Lee Z, Corrado RR, Ogloff JRP. **Women Inmates' Mental Health Needs: Evidence of the Validity of the Jail Screening Assessment Tool (JSAT)**. *The International Journal of Forensic Mental Health* 2004, **3**(2):167-184.

136. O'Neill ML, Nenzel ME, Caldwell W. **Intrusive thoughts and psychopathy in a student and incarcerated sample**. *Journal of Behavior Therapy & Experimental Psychiatry* 2009, **40**(1):147-157.

137. Office of the Correctional Investigator. **Risky Business: An Investigation of the Treatment and Management of Chronic Self-Injury Among Federally Sentenced Women.** <http://www.oci-bec.gc.ca/cnt/rpt/pdf/oth-aut/oth-aut20130930-eng.pdf>. 2013. Accessed.

138. Office of the Correctional Investigator. **A Three Year Review of Federal Inmates Suicides (2011-2014).** <http://www.oci-bec.gc.ca/cnt/rpt/pdf/oth-aut/oth-aut20140910-eng.pdf>. 2014. Accessed.

139. Parker JD, Shaughnessy PA, Wood LM, Majeski SA, Eastabrook JM. **Cross-cultural alexithymia: validity of the 20-item Toronto Alexithymia Scale in North American aboriginal populations**. *Journal of psychosomatic research* 2005, **58**(1):83-88.

140. Penner Hutton K. **Deliberate self-harm in an incarcerated population of youth: An examination of prevalence rates, risk, and protective factors**. *Dissertation Abstracts International* 2011, **74(4-B**(E):Sefe.

141. Perrault S. **Admissions to adult correctional services in Canada, 2011/2012.** [http://www.statcan.gc.ca/pub/85-002-x/2014001/article/11918-eng.htm - a2](http://www.statcan.gc.ca/pub/85-002-x/2014001/article/11918-eng.htm#a2). 2014. Accessed December 14 2014.

142. Perrault S. **Admissions to youth correctional services in Canada, 2011/2012.** <http://www.statcan.gc.ca/pub/85-002-x/2014001/article/11917-eng.htm?fpv=2693>. 2014. Accessed September 22 2014.

143. Picheca JE. **Perpetrators of sexual violence within intimate relationships: Sexual offenders or male batterers?** *Dissertation Abstracts International* 2006, **68**(1-B):631.

144. Plourde C, Brochu S. **Drugs in prison: a break in the pathway**. *Substance use & misuse* 2002, **37**(1):47-63.

145. Plourde C, Brochu S. **Medication and drug use during incarceration: Homeostasis of a setting**. *International Medical Journal* 2002, **9**(3):163-168.

146. Plourde C, Brochu S. **Drugs and alcohol in prison: Examination of the situation in Quebec federal penitentiaries**. *Canadian Journal of Criminology; Corrections, Canadian Journal of Criminology and Criminal Justice* 2002, **44**(2):209-240.

147. Plourde C, Brochu S, Gendron A, Brunelle N. **Pathways of substance use among female and male inmates in Canadian federal settings**. *The Prison Journal* 2012, **92**(4):506-524.

148. Porter S, Birt AR, Boer DP. **Investigation of the criminal and conditional release profiles of Canadian federal offenders as a function of psychopathy and age**. *Law Hum Behav* 2001, **25**(6):647-661.

149. Porter S, Woodworth M. **"I'm sorry I did it... but he started it": a comparison of the official and self-reported homicide descriptions of psychopaths and non-psychopaths**. *Law & Human Behavior* 2007, **31**(1):91-107.

150. Wilson RJ, Abracen J, Looman J, Picheca JE, Ferguson M. **Pedophilia: an evaluation of diagnostic and risk prediction methods**. *Sexual Abuse: Journal of Research & Treatment* 2011, **23**(2):260-274.

151. Thompson J, Zakaria D, Grant B. **Summary of the 2007 National Inmate Infectious Diseases and Risk-Behaviours Survey for Women.** <http://www.csc-scc.gc.ca/research/005008-0238-eng.shtml>. 2011. Accessed June 16 2014.

152. Thompson J, Zakaria D, Grant B. **Aboriginal Men: A Summary of the Findings of the 2007 National Inmate Infectious Diseases and Risk-Behaviours Survey, R-237.** <http://www.csc-scc.gc.ca/research/005008-0237-eng.shtml>. 2011. Accessed June 16 2014.

153. Zakaria D, Thompson JM, Jarvis A, Borgatta F. **Summary of Emerging Findings from the 2007 National Inmate Infectious Diseases and Risk-Behaviours Survey.** <http://www.csc-scc.gc.ca/005/008/092/005008-0211-01-eng.pdf>. 2010. Accessed June 16 2014.

154. Zakaria D, Thompson J, Jarvis A, Smith J. **Testing and Treatment for Human Immunodeficiency Virus and Hepatitis C Virus Infections Among Canadian Federal Inmates, R-223.** <http://www.csc-scc.gc.ca/research/005008-0223-eng.shtml>. 2010. Accessed June 16 2014.

155. Zakaria D. **Relationships between health risk-behaviours, self-perceived risk for infection, and testing for human immunodeficiency virus and hepatitis C virus infections among Canadian federal inmates.** <http://www.csc-scc.gc.ca/research/005008-0254-eng.shtml>. 2011. Accessed June 16 2014.

156. Zakaria D. **Relationships between lifetime health risk-behaviours and self-reported human immunodeficiency virus and hepatitis C virus infection status among Canadian federal inmates.** <http://www.publicsafety.gc.ca/lbrr/archives/cn21491-eng.pdf>. 2012. Accessed June 16 2014.

157. Power J, Brown SL, Usher AM. **Prevalence and incidence of nonsuicidal self-injury among federally sentenced women in Canada**. *Criminal Justice and Behavior* 2013, **40**(3):302-320.

158. Power J, Usher A. **A Descriptive Analysis of Self-injurious Behaviour in Federally Sentenced Women.** <http://www.csc-scc.gc.ca/research/005008-0251-eng.shtml>. 2011. Accessed June 17, 2014.

159. Power J, Usher A. **Correlates and Trajectories to Self-injurious Behaviour in Federally Sentenced Women, R-245.** <http://www.csc-scc.gc.ca/research/005008-0245-eng.shtml>. 2011. Accessed June 18, 2014.

160. Power J. **Non-suicidal self-injury in federally sentenced women: Prevalence, nature, motivations, and pathways**. *Dissertation Abstracts International* 2011, **73**(4-B):2515.

161. Power J, Gordon A, Sapers J, Beaudette J. **A replication study of self-injury incidents in CSC institutions over a thirty-month period, R293.** <http://www.csc-scc.gc.ca/research/005008-0293-eng.shtml>. 2012. Accessed June 16, 2014.

162. Power J, Usher A, Beaudette J. **Self-Injurious Behaviour in Male Offenders: A Multi-Method Investigation, R270.** <http://www.csc-scc.gc.ca/research/005008-0270-eng.shtml>. 2012. Accessed June 17, 2014.

163. Power J, Usher, A. **Correlates and trajectories to self-injurious behaviour in federally sentenced men.** <http://www.publicsafety.gc.ca/lbrr/archives/cn21461-eng.pdf>. 2011. Accessed June 17, 2014.

164. Power J, Riley DL. **A Comparative Review of Suicide and Self-Injury Investigative Reports in a Canadian Federal Correctional Population.** <http://www.csc-scc.gc.ca/005/008/005008-0221-01-eng.shtml>. 2010. Accessed December 11, 2014.

165. Public Safety Canada. **2011 Corrections and Conditional Release Statistical Overview.** [http://www.publicsafety.gc.ca/cnt/rsrcs/pblctns/2011-ccrs/index-eng.aspx - c17](http://www.publicsafety.gc.ca/cnt/rsrcs/pblctns/2011-ccrs/index-eng.aspx#c17). 2011. Accessed December 11, 2014.

166. Public Safety Canada. **Corrections and Conditional Release: Statistical Overview.** <http://www.publicsafety.gc.ca/cnt/rsrcs/pblctns/crrctns-cndtnl-rls-2013/crrctns-cndtnl-rls-2013-eng.pdf>. 2013. Accessed November 25, 2014.

167. Turner NE, Preston DL, Saunders C, McAvoy S, Jain U. **The relationship of problem gambling to criminal behavior in a sample of Canadian male federal offenders**. *Journal of Gambling Behavior* 2009, **25**(2):153-169.

168. Public Safety Canada. **2010 Corrections and Conditional Release Statistical Overview.** [http://www.publicsafety.gc.ca/cnt/rsrcs/pblctns/2010-ccrs/index-eng.aspx - c17](http://www.publicsafety.gc.ca/cnt/rsrcs/pblctns/2010-ccrs/index-eng.aspx#c17). 2010. Accessed December 14, 2014.

169. Public Safety Canada. **2012 Corrections and Conditional Release Statistical Overview.** [http://www.publicsafety.gc.ca/cnt/rsrcs/pblctns/2012-ccrs/index-eng.aspx - c6](http://www.publicsafety.gc.ca/cnt/rsrcs/pblctns/2012-ccrs/index-eng.aspx#c6). 2012. Accessed December 14, 2014.

170. Public Safety Canada. **Corrections and Conditional Release Statistical Overview 2007.** <http://www.publicsafety.gc.ca/cnt/rsrcs/pblctns/2007-ccrs/2007-ccrs-eng.pdf>. 2007. Accessed December 11, 2014.

171. Public Safety Canada. **2008 Corrections and Conditional Release Statistical Overview.** [http://www.publicsafety.gc.ca/cnt/rsrcs/pblctns/2008-ccrs/index-eng.aspx - c10](http://www.publicsafety.gc.ca/cnt/rsrcs/pblctns/2008-ccrs/index-eng.aspx#c10). 2008. Accessed December 11, 2014.

172. Public Safety Canada. **2009 Corrections and Conditional Release Statistical Overview.** [http://www.publicsafety.gc.ca/cnt/rsrcs/pblctns/2009-ccrs/index-eng.aspx - c10](http://www.publicsafety.gc.ca/cnt/rsrcs/pblctns/2009-ccrs/index-eng.aspx#c10). 2009. Accessed December 14, 2014.

173. Reckdenwald A, Mancini C, Beauregard E. **The cycle of violence: examining the impact of maltreatment early in life on adult offending**. *Violence and victims* 2013, **28**(3):466-482.

174. Rezansoff SN, Moniruzzaman A, Gress C, Somers JM. **Psychiatric diagnoses and multiyear criminal recidivism in a Canadian provincial offender population**. *Psychology, Public Policy, and Law* 2013, **19**(4):443-453.

175. Robinson D, Mirabelli, L. **Summary of Findings of the 1995 CSC National Inmate Survey.** <http://www.csc-scc.gc.ca/research/b14e-eng.shtml>. 1996. Accessed November 18 2014.

176. Rothon DA, Strathdee SA, Cook D, Cornelisse PG. **Determinants of HIV-related high risk behaviours among young offenders: a window of opportunity**. *Canadian Journal of Public HealthRevue Canadienne de Sante Publique* 1997, **88**(1):14-17.

177. Sapers H. **Annual Report of the Office of the Correctional Investigator 2012-2013.** <http://www.oci-bec.gc.ca/cnt/rpt/pdf/annrpt/annrpt20122013-eng.pdf>. 2013. Accessed.

178. Sapers H. **Annual Report of the Correctional Investigator 2009-2010.** <http://www.oci-bec.gc.ca/cnt/rpt/pdf/annrpt/annrpt20092010-eng.pdf>. 2010. Accessed November 25, 2014.

179. Sapers H. **Annual Report of The Office of the Correctional Investigator 2010-2011.** <http://www.oci-bec.gc.ca/cnt/rpt/pdf/annrpt/annrpt20102011-eng.pdf>. 2011. Accessed December 14, 2014.

180. Sapers H. **Annual Report of the Office of the Correctional Investigato 2011-2012.** <http://www.oci-bec.gc.ca/cnt/rpt/pdf/annrpt/annrpt20112012-eng.pdf>. 2012. Accessed December 14, 2014.

181. Scheim A, Cherian M, Bauer G, Zong X. **Joint Effort: Prison Experiences of Trans PULSE Participants and Recommendations for Change.** <http://www.pasan.org/Toolkits/Trans_PULSE_Project_-_Prison_Experiences-EN.pdf>. 2013. Accessed July 31 2014.

182. Smith A, Cox K, Poon C, Stewart D, McCreary Centre Society. **Time Out III: A profile of BC youth in custody.** <http://www.mcs.bc.ca/pdf/Time_Out_III.pdf>. 2013. Accessed December 1 2014.

183. Stewart L, Gabora N, Kropp R, Lee Z. **Family violence programming: Treatment outcome for Canadian federally sentenced offenders.** <http://www.csc-scc.gc.ca/research/r174-eng.shtml>. 2008. Accessed December 11, 2014.

184. Stewart LA, Gabora N, Kropp PR, Lee Z. **Effectiveness of risk-needs-responsivity-based family violence programs with male offenders**. *Journal of Family Violence* 2014, **29**(2):151-164.

185. Stewart L, Wilton G. **Validation of the Computerised Mental Health Intake Screening System (CoMHISS) in a Federal Male Offender Population. .** <http://www.csc-scc.gc.ca/research/005008-0244-eng.shtml>. 2011. Accessed July 2, 2014.

186. Stewart L, Harris A, Wilton G, Archambault K, Cousineau C, Varrette S, Power J. **An Initial Report on the Results of the Pilot of the Computerized Mental Health Intake Screening System.** <http://www.csc-scc.gc.ca/research/005008-0218-01-eng.shtml>. 2010. Accessed December 11, 2014.

187. Stewart L, Wilton G. **Federally sentenced offenders with mental disorders: Correctional outcomes and correctional response, R268**. In*.*: Correctional Service of Canada; 2012.

188. Sun F, Cousineau M, Brochu S, White ND. **Consumption of psychoactive substances and seriousness of crime**. *Canadian Journal of Criminology, Canadian Journal of Criminology &amp; Corrections* 2004, **46**(1):1-26.

189. Swihart G. **Female offenders: Attachment and parenthood**. *Dissertation Abstracts International* 2004, **64**(10-B):5266.

190. The McCreary Centre Society. **Time Out: A Profile of BC Youth in Custody**. 2001.

191. Wichmann C, Serin R, Motiuk L. **Predicting Suicide Attempts Among Male Offenders in Federal Penitentiaries.** <http://www.csc-scc.gc.ca/research/r91-eng.shtml>. 2000. Accessed June 19, 2014.

192. Wichmann C, Serin R, Abracen J. **Women Offenders Who Engage in Self-harm: A Comparative Investigation.** <http://www.csc-scc.gc.ca/research/r123-eng.shtml>. 2002. Accessed June 19, 2014.

193. Todd KL. **A neuropsychological study of traumatic brain injury among a Canadian sample of male federal offenders**. *Dissertation Abstracts International* 2010, **71**(11-B):7069.

194. Trevethan S, Auger S, Moore JP. **The effect of family disruption on Aboriginal and non-Aboriginal inmates.** <http://www.csc-scc.gc.ca/research/r113-eng.shtml>. 2001. Accessed January 13, 2015.

195. Turnbull SD. **Personality pathology and adult attachment in female offenders**. *Dissertation Abstracts International* 1996, **58**(4-B):2170.

196. Turner NE, Preston DL, McAvoy S, Gillam L. **The use of tobacco as gambling currency by federal offenders in Canada before and after a tobacco ban**. *Journal of Gambling Behavior* 2013, **29**(1):97-107.

197. Ulzen TP, Hamilton H. **The nature and characteristics of psychiatric comorbidity in incarcerated adolescents**. *Canadian Journal of Psychiatry - Revue Canadienne de Psychiatrie* 1998, **43**(1):57-63.

198. Young SJ, Longstaffe S, Tenenbein M. **Inhalant abuse and the abuse of other drugs**. *American Journal of Drug &amp; Alcohol Abuse* 1999, **25**(2):371-375.

199. Ulzen T, Hamilton H. **Post Traumatic Stress Disorder in Incarcerated Adolescents**. *The Canadian Child and Adolescent Psychiatry Review* 2003, **12**(4):113-116.

200. Usher AM, Stewart LA, Wilton G. **Attention deficit hyperactivity disorder in a Canadian prison population**. *International Journal of Law &amp; Psychiatry* 2013, **36**(3-4):311-315.

201. Valliant PM, Clark LM. **An evaluation of nonassaultive, assaultive, and sexually assaultive adolescents at pretrial sentencing: A comparison on cognition, personality, aggression, and criminal sentiments**. *Psychological reports* 2009, **105**(3, Pt2):1077-1091.

202. Valliant PM, De Wit M, Bowes R. **Cognitive and personality factors associated with assaultive and domestic offenders**. *Psychological reports* 2004, **94**(3,Pt2):1180-1184.

203. Valliant PM, Freeston A, Pottier D, Kosmyna R. **Personality and executive functioning as risk factors in recidivists**. *Psychological reports* 2003, **92**(1):299-306.

204. Valliant PM, Gauthier T, Pottier D, Kosmyna R. **Moral reasoning, interpersonal skills, and cognition of rapists, child molesters, and incest offenders**. *Psychological reports* 2000, **86**(1):67-75.

205. Valliant PM, Gristey C, Pottier D, Kosmyna R. **Risk factors in violent and nonviolent offenders**. *Psychological reports* 1999, **85**(2):675-680.

206. Viljoen JL, O'Neill ML, Sidhu A. **Bullying behaviors in female and male adolescent offenders: Prevalence, types, and association with psychosocial adjustment**. *Aggressive Behavior* 2005, **31**(6):521-536.

207. Vincent GM, Vitacco MJ, Grisso T, Corrado RR. **Subtypes of Adolescent Offenders: Affective Traits and Antisocial Behavior Patterns**. *Behavioral Sciences and the Law* 2003, **21**(6):695-712.

208. Wanklyn SG, Day DM, Hart TA, Girard TA. **Cumulative childhood maltreatment and depression among incarcerated youth: Impulsivity and hopelessness as potential intervening variables**. *Child maltreatment* 2012, **17**(4):306-317.

209. Weekes J, Moser A, Ternes M, Kunic D. **Substance Abuse Among Male Offenders.** <http://www.csc-scc.gc.ca/research/rs09-02-eng.shtml>. 2009. Accessed July 30, 2014.

210. Zinger I. **The psychological effects of 60 days in administrative segregation. (solitary confinement, prisoners)**. *Dissertation Abstracts International* 1999, **60**(6-B):2932.

211. Zinger I, Wichmann C, Andrews DA. **The psychological effects of 60 days in administrative segregation**. *Canadian Journal of Criminology & Corrections, Canadian Journal of Criminology and Criminal Justice* 2001, **43**(1):47-83.

212. Burd L, Selfridge RH, Klug MG, Juelson T. **Fetal alcohol syndrome in the Canadian corrections system**. *Journal of Fetal Alcohol Syndrome International* 2003, **1**:1-9.

213. Sapers H. **Annual Report of the Correctional Investigator: 2013-2014.** <http://www.oci-bec.gc.ca/cnt/rpt/pdf/annrpt/annrpt20132014-eng.pdf>. 2014. Accessed November 25 2014.

214. Antonowicz D, Winterdyk J. **A review of deaths in custody in three Canadian provinces**. *Canadian Journal of Criminology and Criminal Justice* 2014:85-103.

215. Dietrich A. **Characteristics of child maltreatment, psychological dissociation, and somatoform dissociation of Canadian inmates**. *Journal of Trauma and Dissociation* 2003, **4**(1):81-100.

216. Dietrich AM. **PTSD and associated features as predictors of revictimization and perpetration with samples of adults abused during childhood**. *Dissertation Abstracts International* 2003, **64**(12-B):6325.

217. Preston DL, McAvoy S, Saunders C, Gillam L, Saied A, Turner NE. **Problem gambling and mental health comorbidity in Canadian federal offenders**. *Criminal Justice and Behavior* 2012, **39**(10):1373-1388.

218. Booth L. **Effective correctional programs for women offenders. Research report R-279.** <http://www.csc-scc.gc.ca/research/005008-0279-eng.shtml>. 2012. Accessed July 25, 2014.

219. Brink JH, Doherty D, Boer A. **Mental disorder in federal offenders: A Canadian prevalence study**. *International journal of law and psychiatry* 2001, **24**(4-5):339-356.
